# Transferability and polymorphism of barley EST-SSR markers used for phylogenetic analysis in *Hordeum chilense*

**DOI:** 10.1186/1471-2229-8-97

**Published:** 2008-09-28

**Authors:** Almudena Castillo, Hikmet Budak, Rajeev K Varshney, Gabriel Dorado, Andreas Graner, Pilar Hernandez

**Affiliations:** 1 Institute for Sustainable Agriculture (IAS-CSIC), Alameda del Obispo s/n, 14080 Córdoba, Spain; 2 Sabanci University, Engineering and Natural Sciences, Biological Science and Bioengineering Program, Orhanli 34956 Tuzla-Istanbul, Turkey; 3 International Crops Research Institute for the Semi-Arid Tropics (ICRISAT), Patancheru – 502 324, Andhra Pradesh, India; 4 Dep. Bioquímica y Biología Molecular, Campus Rabanales, C6-1-E17, Universidad de Córdoba, 14071 Córdoba, Spain; 5 Leibniz Institute of Plant Genetics and Crop Plant Research (IPK), Corrensstrasse 3, D-06466 Gatersleben, Germany

## Abstract

**Background:**

*Hordeum chilense*, a native South American diploid wild barley, is a potential source of useful genes for cereal breeding. The use of this wild species to increase genetic variation in cereals will be greatly facilitated by marker-assisted selection. Different economically feasible approaches have been undertaken for this wild species with limited direct agricultural use in a search for suitable and cost-effective markers. The availability of Expressed Sequence Tags (EST) derived microsatellites or simple sequence repeat (SSR) markers, commonly called as EST-SSRs, for barley (*Hordeum vulgare*) represents a promising source to increase the number of genetic markers available for the *H. chilense *genome.

**Results:**

All of the 82 barley EST-derived SSR primer pairs tested for transferability to *H. chilense *amplified products of correct size from this species. Of these 82 barley EST-SSRs, 21 (26%) showed polymorphism among *H. chilense *lines. Identified polymorphic markers were used to test the transferability and polymorphism in other Poaceae family species with the aim of establishing *H. chilense *phylogenetic relationships. *Triticum aestivum*-*H. chilense *addition lines allowed us to determine the chromosomal localizations of EST-SSR markers and confirm conservation of the linkage group.

**Conclusion:**

From the present study a set of 21 polymorphic EST-SSR markers have been identified to be useful for diversity analysis of *H. chilense*, related wild barleys like *H. murinum*, and for wheat marker-assisted introgression breeding. Across-genera transferability of the barley EST-SSR markers has allowed phylogenetic inference within the Triticeae complex.

## Background

Wild species constitute a potential source of genetic variation for cultivated species. Besides, they can be analyzed to answer the long-lasting questions concerning the origins, evolution and spread of major agricultural crops of the world. Recently, there has been considerable progress in plant genomics, leading to novel molecular breeding tools to reduce the costs and to simplify the assays. Plant genome research has been focused on the major crops and model species and a vast amount of genomic information has been accumulated. This information will provide an opportunity to use it as sources of information for thousands of minor grass species [1].

*Hordeum chilense *Roem et Schultes is a native South American diploid perennial wild barley (2n = 2x = 14), included in the section Anisolepsis [2]. It belongs to a heterogeneous group of South American *Hordeum *species and it is one of the species of the genus *Hordeum *with a high potential for cereal breeding purposes, given its high crossability with other members of the Triticeae tribe. *H. chilense *was used to obtain fertile amphiploids with wheat of different ploidy levels (diploid, tetraploid and hexaploid). These amphiploids were named *Tritordeum *and are the basic genetic material for using *H. chilense *genetic variability in wheat breeding [3]. *H. chilense *has agronomically interesting characteristics, like high carotenoid content, biotic and abiotic stress resistance, and variability for seed storage proteins [4]. A new cytoplasmic male sterility (CMS) source in bread wheat using the cytoplasm of *H. chilense *has also been reported [5].

The characterization of genetic variability in wild species like *H. chilense *and the development of tools to introduce it into cultivated crops are important plant breeding goals. The analysis of DNA sequence variation is of major importance in genetic studies. In this context, molecular markers are useful tools for assaying genetic variation, and have greatly enhanced the genetic analysis of crop plants. High development costs make it impractical to develop molecular markers directly from wild species like *H. chilense*. However, if markers developed in related crop species can be used, genetic analysis of *H. chilense *could be advanced rapidly [6].

Several types of molecular markers, including Random Amplified Polymorphic DNA (RAPD) and Sequence Characterized Amplified Regions (SCARs) [7,8] have been developed *de novo *for *H. chilense*. Comparative genetic analysis has been established in the grass genomes [9] showing significant conservation of marker and gene order across the cereal crops. On this basis, Sequence Tagged Sites (STSs) and microsatellites or simple sequence repeats (SSRs) were transferred from wheat and barley to *H. chilense *[10,11]. The SSR markers have been the molecular markers of choice for molecular breeders due to their codominant inheritance, multi-allelic nature and easy detection [12]. Recent studies have used the increase on the availability of large numbers of Expressed Sequence Tags (ESTs) in public databases, to the search for SSRs present in ESTs i.e. in barley [13], sugarcane [14], bread wheat [15], apricot and grape [16], rice [17] and *Vaccinium *[18].

Across-species transferability of SSRs derived from EST databases is greater than that of SSRs derived from enriched genomic DNA libraries, as they originate from expressed regions and therefore they are more conserved across a number of related species than non-coding regions [19]. They have shown to be useful for comparative mapping across species, comparative genomics, and evolutionary studies and they have been shown to posses a higher potential for inter-specific transferability than genomic SSRs [15,16,20-23]. On the other hand, they are expected to be less polymorphic within the species due to its conserved nature [19]. In summary, EST-SSR has provided a valuable source of new PCR-based molecular markers in cereal crops.

We have tested the transferability of 82 EST-SSR markers developed in barley [24,25], and their potential use as new molecular tools for introgression, variability and phylogenetic analysis of the *H. chilense *genome. The chromosomal locations of the transferred and polymorphic EST-SSRs were assigned using wheat chromosome addition lines [26].

The grass family (Poaceae) is formed by 600 genera and between 9,000 to 10,000 species of grasses. This family comprises the most important cultivated crops like wheat, barley, rye, and rice [27]. *H. chilense *belongs to the Poaceae family, Triticeae tribe, genus *Hordeum*. Previous cytogenetic work suggested that *H. chilense *chromosomes are more similar to the D- than to the A- or B-genomes of wheat [28]. The phylogenetic relationships of *H. chilense *with respect to *Triticum *and *Hordeum *have not been studied in detail so far. For this reason, the set of EST-SSR primers that successfully amplified *H. chilense *and showed polymorphism was further tested for transferability to other species of the Poaceae family with the aim to investigate phylogenetic relationships.

## Methods

### Plant material and DNA extraction

Two accessions (H1 and H7) of *H. chilense *and one genotype ('Barke') of *H. vulgare *were used for the initial transferability analysis of 82 barley EST-derived SSR-markers. Barke was included as standard because this cultivar was used to construct the EST libraries screened to search for SSRs [29]. A set of wheat (cv. 'Chinese Spring')/*H. chilense *accession H1 addition lines [26] and their wheat and wild barley donors were used for the chromosome location of the 21 transferred and polymorphic EST-SSR markers. For the phylogenetic analysis, two hexaploid wheat (*Triticum aestivum *L.) accessions ('T21' cv. 'Chinese Spring' and 'T20'), two tetraploid wheat (*T. durum*) accessions ('T22' and 'T81' cv. 'Yavaros'), the diploid wheat (*T. tauschii*) accession ('T6'), two *T. urartu *accessions ('T485' and 'T486'), two barley (*H. vulgare*) cultivars ('Betzes' and 'Barke'), two *H. chilense *accessions (H1 and H7), one *H. murinum *and two *Brachypodium distachyon *L. accessions ('Bd1' and 'Bd6') were evaluated.

Total genomic DNA was isolated from young frozen leaf tissue using the CTAB method of Murray and Thompson [30] as modified by Hernandez et al. [31]. The concentration of each sample was estimated by comparing band intensity with lambda DNA digests of known concentrations under UV light, after 0.8% (w/v) agarose gel electrophoresis and ethidium bromide staining.

### Amplification and transferability of barley EST-SSR markers

A set of 82 barley EST-derived SSR markers developed by Varshney et al. [25] and uniformly distributed across the *H. vulgare *chromosomes were tested for amplification of the *H. chilense *DNA from lines H1 and H7 using *H. vulgare *cv. 'Barke' as control. For each EST-SSR, the forward primer was labeled with one fluorescent dye for detection on an ABI Prism 310 Genetic Analyzer from Applied Biosystems (Foster City, CA, USA). The PCR amplification was carried out using a GeneAmp PCR System 9700 in 20 μl reactions consisting of a 1 × PCR buffer including 1.5 μM MgCl_2_, 200 μM dNTPs, 250 nM of each primer, 0.25 U of *Taq *Gold DNA polymerase (PCR cycler and reagents from Applied Biosystems), and 20 ng of genomic DNA. PCR conditions followed a touch-down protocol as described in [24] and [25]: an initial denaturing step of 10 min at 94°C was followed by 45 cycles with denaturation at 94°C for 30 s and extension at 72°C for 30 s, respectively. The annealing temperature was decreased in 0.5°C per cycle, from 60°C in the first cycle to 55°C after the 10th cycle, and was then kept constant for the remaining 35 cycles (always 30 s). After 45 cycles, a final extension step was performed at 72°C for 5 min.

**Figure 1:**
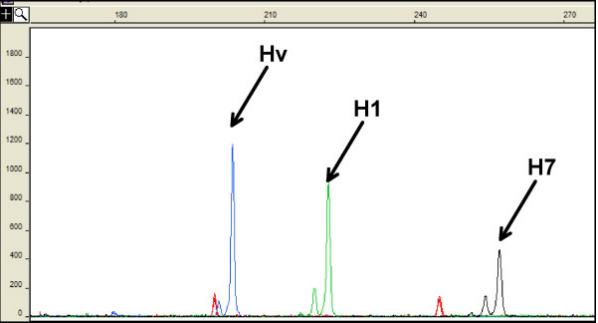
**Polymorphism in *Hordeum chilense *lines detected by M7.8 (barley GBM1464) marker of linkage group 7H**. Electropherogram obtained on an ABI Prism 310 Genetic Analyzer, showing the polymorphism. H1 and H7: *H. chilense *lines. Hv: *H. vulgare *cv. 'Betzes'.

**Figure 2:**
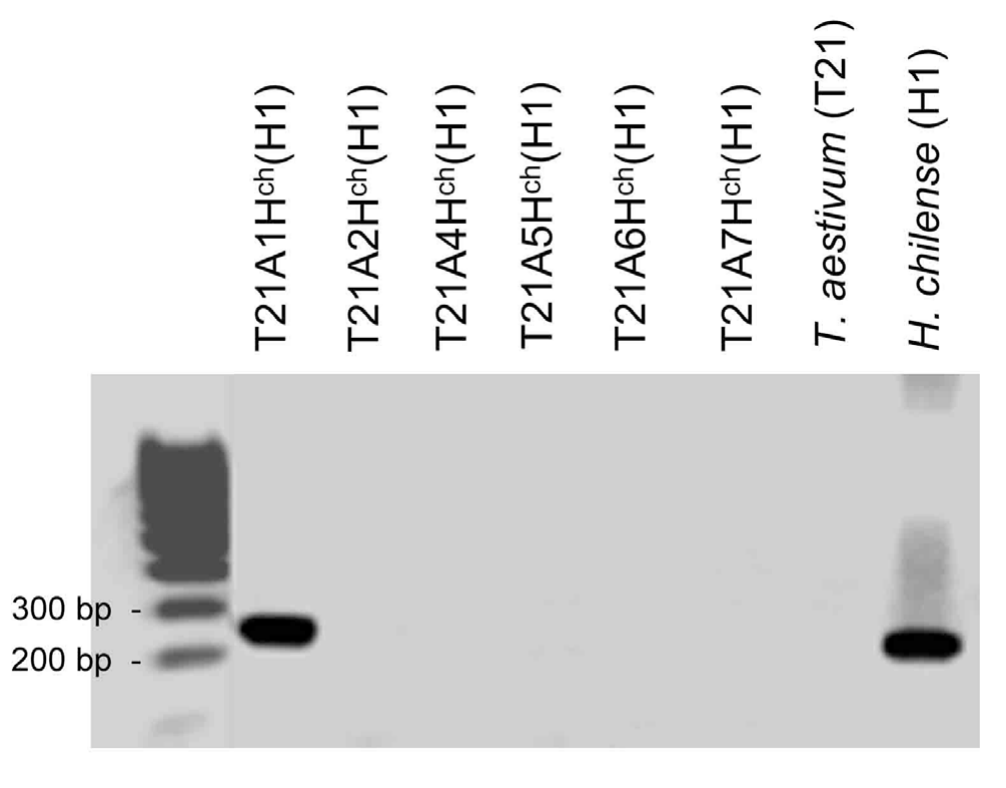
**PCR amplification of marker M1.2 (barley GBM 1029) marker (1H) in *Hordeum chilense *lines**. The PCR amplification products were segregated on agarose gel electrophoresis and visualized under UV light in the presence of ethidium bromide. Lanes (left to right): *T. aestivum *accession T21 – *H. chilense *accession H1 addition lines (1Hch + telo, 2Hchα, 4Hch, 5Hch, 6Hch, and 7Hch), wheat T21 parent and *H. chilense *H1 parent.

**Figure 3:**
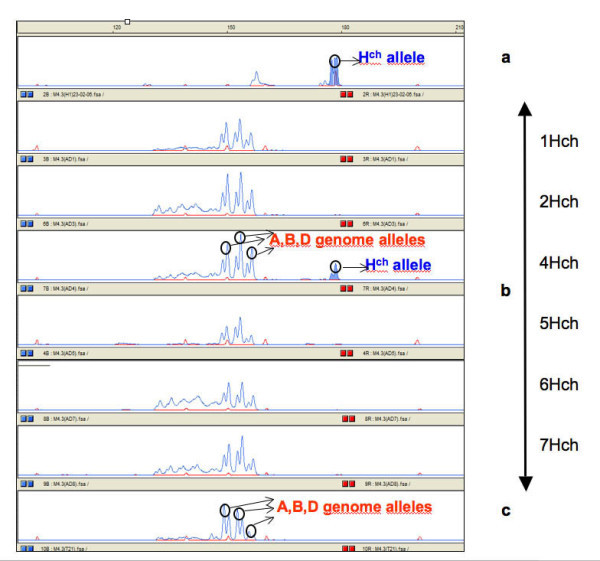
**Amplification profiles of marker M4.3 (barley GBM1067) marker (4H) in the genomes of *Hordeum chilense*, *Triticum aestivum *and disomic chromosome addition lines on the ABI 310 capillary system**. (a) *H. chilense*, H1 line; (b) *T. aestivum *accession T21 – *H. chilense *accession H1 addition lines (1Hch + telo, 2Hchα, 4Hch, 5Hch, 6Hch, and 7Hch); (c) *T. aestivum *accession T21.

Amplification products derived from fluorescently labeled primers were resolved by capillary electrophoresis on the ABI Prism 310 Genetic Analyzer. The fragment sizes were calculated using the computer program GeneScan from the same manufacturer, by comparison with an internal size standard (Figure 1). The presence and pattern of stuttering was locus-specific and was analyzed as an indication of correct locus transferability. For instance, Figure 1 shows the amplification of a barley trinucleotide EST-SSR, with weak -3 bp stutters. The stutters are also present in *H. chilense *lines H1 and H7, with a similar pattern (weak -3 bp stutters, as shown in Figure 1). A selection of primer pairs that showed polymorphism in *H. chilense *lines were tested in the other species using the same PCR conditions. To assign barley EST-SSR markers to *H. chilense *chromosomes, six *T. aestivum*/*H. chilense *accession H1 addition lines including a monotelodisomic 1H^ch^S addition, a ditelosomic addition for 2H^ch ^alpha arm, and disomic addition lines for chromosomes 4H^ch^, 5H^ch^, 6H^ch ^and 7H^ch ^were used [26] (Figures 2 and 3). The addition line carrying the chromosome 3 is not available and therefore the assignment of markers to this chromosome was done using the lack of amplification in the other addition lines mentioned above.

### Phylogenetic data analysis

**Figure 4:**
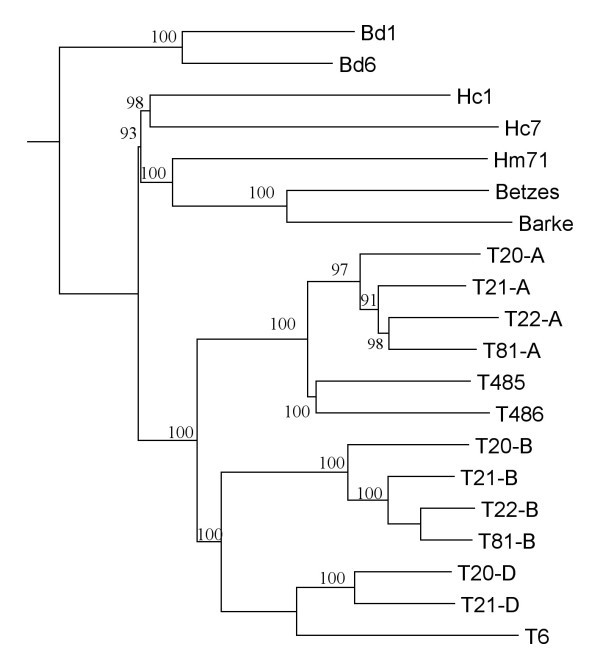
**Consensus tree of species reconstructed from 1000 NJ trees obtained from data resampled in a set of 13 EST-SSR markers**. The accession codes are: Hc1 and Hc7, *H. chilense*; Hm71, *H. murinum*; Betzes and Barke, *H. vulgare*; T20-A, T20-B and T20-D, *T. aestivum *A- B- and D-genomes; T21-A, T21-B and T21-D, *T. aestivum *cv. 'Chinese Spring' A- B- and D-genomes; T22-A and T22-B, *T. durum *A- and B-genomes; T81-A and T81-B, *T. durum *cv. 'Yavaros'; T485 and T486, *T. urartu*; T6, *T. tauschii*; Bd1 and Bd6, *B. distachyon*. The numbers at the nodes indicate the percentage number of 1.000 bootstrap replications.

A binary matrix was generated, where the presence or absence of each allele was coded by 1 or 0 respectively. The binary data were used to calculate the distance matrix using the Jaccard's similarity coefficient [32], because the binary information was asymmetric (the shared absence of a given allele did not contribute to genetic similarity, as no null alleles were found). The software package Phylip [33] was used to calculate the genetic relationships by neighbor-joining (NJ) analysis. The reliability and goodness of fit of the dendrogram obtained was tested by bootstrap analysis based on 1,000 permutations, followed by the program Consense module in the software package Phylip. The dendrogram (Figure 4) was constructed using the NJ method with the program TreeView [34]. The tree was rooted using *B. distachyon *as outgroup. For the phylogenetic analysis, hexaploid wheat allelic data were separated into its three A, B and D genome alleles, and tetraploid wheat allelic data were separated into its two A and B genome alleles (Figure 4). A second dendrogram (not shown, as the relationships are similar) was obtained only for the species belonging to the Triticeae tribe, using the UPGMA clustering method, as the divergence between these species was quite recent and therefore the molecular clock could be assumed.

## Results

### Amplification and polymorphism of barley EST-SSR markers

All eighty-two sets of primer pairs from barley EST-SSR markers amplified products within the *H. chilense *genome. The amplification patterns of 28 EST-SSRs were not reliable though. An amplification pattern was considered as not reliable for any of the following four reasons: i) the presence of too many amplification products; ii) the presence of faint bands; iii) the molecular weight of the *H. chilense *amplification product was of a different size range than the *H. vulgare *molecular weight; iv) the *H. vulgare *stuttering profile was not maintained in *H. chilense*. Of the remaining markers, twenty-one primer pairs (26%) showed polymorphism between H1 and H7 *H. chilense *lines (Figure 1) and were renamed according to their chromosomal location in *H. chilense *(Table 1). These twenty-one primer pairs were tested in the rest of species included in this study (Table 1). From them, 100% amplified in the *H. murinum *genome; 19 (90%) amplified in the *T. durum *genome; 17 (81%) amplified the genomes of *T. aestivum*, *T. urartu *and *T. tauschii*, and 16 (76%) amplified the genome of *B. distachyon*. Primers for three EST-SSR markers (M1.4, M4.1 and M5.6) amplified only one of the two *B. distachyon *accessions tested. Similarly, amplification of the primer pair M1.8 was observed only in one of the two *T. urartu *and *T. durum *accessions tested. Primers for 11 EST-SSR markers (M1.2, M2.6, M3.7, M4.1, M4.3, M4.12, M6.5, M6.7, M6.8, M7.8 and M7.9) were monomorphic for any individual species. Five primer pairs detected polymorphism within *T. aestivum*, four within *T. durum*, four within *T. urartu *and six within *B. distachyon*. All of the 21 primer pairs tested detected interspecies polymorphisms.

**Table 1:** Intergeneric amplification of selected barley EST-SSRs.

*H. chilense *marker	*H. vulgare *marker	Chromosome	*H. chilense*	*H. vulgare*	*H. murinum*	*T. durum*	*T. tauschii*	*T. urartu*	*T. aestivum*	*B. distachyon*
M1.2*	GBM1029	1	+	+	+	+	+	+	+	+
M1.4	GBM1002	1	+	+	+	+	+	+	+	+
M1.8	GBM1411	1	+	+	+	+	+	+	-	-
M2.5	GBM1047	2	+	+	+	+	+	+	-	+
M2.6*	GBM1036	2	+	+	+	+	+	+	+	+
M2.11	GBM1462	2	+	+	+	-	-	-	-	-
M3.7*	GBM1069	3	+	+	+	+	+	+	+	+
M4.1*	GBM1055	4	+	+	+	+	+	+	+	+
M4.3*	GBM1067	4	+	+	+	+	+	+	+	+
M4.4*	GBM1020	4	+	+	+	+	+	+	+	+
M4.10*	GBM1465	4	+	+	+	+	+	+	+	+
M4.12*	GBM1323	4	+	+	+	+	+	+	+	+
M4.15*	GBM1350	4	+	+	+	+	+	+	+	+
M5.6*	GBM1064	5	+	+	+	+	+	+	+	+
M6.5*	GBM1008	6	+	+	+	+	+	+	+	+
M6.7	GBM1076	6	+	+	+	+	-	-	+	-
M6.8	GBM1400	6	+	+	+	+	+	-	+	-
M7.1	GBM1060	7	+	+	+	+	-	+	+	-
M7.4*	GBM1058	7	+	+	+	+	+	+	+	+
M7.8	GBM1464	7	+	+	+	+	+	+	-	+
M7.9*	GBM1432	7	+	+	+	+	+	+	+	+

+ Amplification

- No amplification

* Markers used for the construction of the dendrogram

### Chromosomal location of barley EST-SSR markers in *H. chilense*

In order to locate polymorphic barley EST-SSR markers onto the *H. chilense *chromosomes, a set of disomic *T. aestivum-H. chilense *addition lines [26] was used. All the primer pairs yielded amplification in the expected homoeologous chromosome (Figures 2 and 3). Amplification products that are present in *H. chilense *but absent in wheat, as well as the amplification products of different size were of particular interest, as these markers can be used for the detection of *H. chilense *chromosomes in the wheat genetic background for introgression analysis.

Four out of 21 SSR markers amplified a SSR fragment in *H. chilense*, but not in wheat and 15 markers amplified PCR products of a different size range in *H. chilense *and wheat. Therefore, a total of 19 markers were found to be suitable for the analysis of *H. chilense*/wheat introgression.

### Phylogenetic relationships

The potential use of the barley EST-SSR markers to infer the phylogenetic relationships among the species studied (*T. aestivum*, *T. durum*, *T. urartu*, *T. tauschii*, *H. vulgare*, *H. murinum*, *H. chilense *and *B. distachyon*) was analyzed using the 13 EST-SSR markers that produced amplification products in all analyzed species. The NJ tree obtained (Figure 4) indicated that all the accessions were clustered according to their genome constitution. The bootstrap values are consistent and they are generally higher than 90%. The *Triticum *species were divided into two groups, one including the D- and B-genome of wheats, and the other including the A-genome of wheat. The *Hordeum *clade was separated into two clusters: one included the *H. chilense *accessions and the other one included *H. vulgare *and *H. murinum *species. *B. distachyon *was used as outgroup to root the NJ tree. The dendrogram obtained by the UPGMA clustering method (not shown) generated similar results to the NJ analysis.

## Discussion

Genomic SSRs have been extensively used for mapping, genetic diversity analysis, and plant breeding, but have a lower rate of transferability across species when compared to the EST-SSR markers. Thus, the latter are a better choice for application in cross-species phylogenetic studies [35], and are also valuable tools for plant breeding, germplasm collection conservation [36], and to measure genetic diversity [37]. More than 500.000 ESTs are presently available for barley and represent an invaluable resource for the development of SSR markers [24,25]. To systematically exploit potentially useful, albeit less studied wild species, like *H. chilense *it would be desirable to use these EST-SSR markers as anchors to the cultivated species genome.

A set of barley genomic SSR markers [38] has been previously tested in *H. chilense *[11]. As expected, the level of transferability (66%) of barley EST-SSR markers found in the present work is higher than for the neutral SSRs (54%). Additionally, the level of polymorphism detected within *H. chilense *with the EST-SSRs (26%) is also higher than with the genomic SSRs (6%).

Our results confirm the high cross-species transferability of the set of 165 barley EST-SSRs tested in wheat, rye and rice [13]. Varshney et al. [13] observed 78.2% amplicons in wheat, 75.2% in rye and 42.4% in rice, demonstrating the high potential of EST-SSR markers for comparative mapping among these species. In the present study, we tested the subset of 21 EST-SSR markers showing higher level of transferability [13] and polymorphism in *H. chilense *lines across genera for phylogenetic inference. We observed 100% amplification of the selected barley EST-SSR markers in the genome of *H. murinum*, 90% amplification in the genome of *T. durum*, 81% amplification in the genomes of *T. aestivum*, *T. urartu *and *T. tauschii *and 76% amplification in the genome of *B. distachyon*. Our results confirm the general observation that the rate of EST-SSRs transferred across species or genera decays as the species or genera are more phylogenetically distant [19].

On the other hand, the sensibly higher transfer rate to *B. distachyon *than to rice confirms the usefulness of *Brachypodium *as a model species for barley and wheat and gave us the species of choice for rooting the phylogenetic tree in the present study. Our results showed a higher transferability of barley EST-SSRs to *H. chilense *than to other wild barley species reported previously [24], where 80% of examined barley markers were successfully amplified in wild barley accessions and about 60% of the primers yielded amplification products in wheat and rye. The high transferability among *Triticeae *species and genera has also been reported for wheat EST-SSR markers. For instance, Tang et al. [39] tested 243 wheat EST-SSR markers, from which 216 (88.9%) produced amplicons in wheat, 211 (86.8%) in barley, 187 (77.0%) in rice and 166 (68.3%) in maize. Zhang et al. [40] reported the transferability of bread wheat EST-SSRs to closely related Triticeae species, ranging from 76.7% for *A. tauschii *to 90.4% for *T. durum*. The rates were lower for more distant relatives such as barley (50.4%) or rice (28.3%). Similar results were obtained by Yu et al. [22], who found that a total of 53% of the wheat EST-SSR markers produced amplicons in barley.

The location of barley EST-SSR on the *H. chilense *chromosomes was determined by the amplification of the available wheat addition lines. They were all found in the same linkage group as barley, thus corroborating the conserved nature of these markers and their potential use in comparative genomics among species. Conserved chromosome locations, together with strong selection criteria (including similar molecular weight range to the donor species and conserved locus stuttering patterns) make these markers' transferability robust enough for their practical application. Fifteen barley EST-SSR produced amplicons of different size in wheat and *H. chilense*, and four did not amplify in wheat. Therefore, 19 barley EST-SSR markers are useful for wheat-*H. chilense *introgression analysis.

Wheat EST-SSRs have been recently demonstrated useful for phylogenetic analysis among the Triticeae species [23]. One of the aims of this study was to infer phylogenetic relationships among the *H. chilense *genome, the cultivated barley genome and the wheat genomes using barley EST-SSRs (Figure 4). In the obtained dendrogram, the nodes were significantly supported by bootstrap analysis, indicating that there were subgroups that could be clearly separated.

As expected, the *H. chilense *genome was situated in the clade of the *Hordeum *species, despite the reported cytogenetic similarity with the D genome of wheat [1]. The species *H. murinum *(Xu-genome) was closer to *H. vulgare *(I-genome) than to *H. chilense *(H-genome). Several other studies have also grouped species of the I- and Xu-genomes [41], using sequences from three nuclear regions DMC1, EF-G and ITS; and the *vrs1 *locus [42].

In the clade of *Triticum *species, the hexaploid and tetraploid wheats allelic data were separated into their genomes. A closer association was observed between the B- and D-genome and tetraploid species were closely related to hexaploid species, which is in agreement with a previous analysis based on wheat EST-SSR data [23].

## Conclusion

Our study shows the utility of barley EST-SSR for the genetic analysis of *H. chilense*, with a remarkably high level of polymorphism within this species. It highlights a reliable and efficient way of obtaining microsatellite markers for wild relatives of a major crop. The transferred markers have shown to be useful for phylogenetic studies among the Triticeae species, and to anchor the *H. chilense *genome within the wheat-barley framework using *Brachypodium *as a root genome. The availability of additional sets of mapped EST-derived SSR markers for barley and other Triticeae genomes will assist the development of molecular maps for *H. chilense *and its integration into the genomic network of grass species.

## Authors' contributions

AC carried out most of the molecular work and drafted the manuscript. RKV, HB, GD and PH were involved in designing and planning the work and interpreting the results. HB, RKV, GD and AG edited the manuscript critically. PH and AG conceived th**e **study. PH coordinated the study and helped to draft the manuscript. All authors have read and approved the final manuscript.

## References

[B1] Wang ML, Barkley NA, Yu JK, Dean RE, Newman ML, Sorrells ME, Pederson G (2005). Transfer of simple sequence repeat (SSR) markers from major cereal crops to minor grass species for germplasm characterization and evaluation. Plant Genetic Resources: characterization and utilization.

[B2] Bothmer R, Jacobsen N, Baden C, Jørgensen RB, Linde-Laursen I (1995). An Ecogeographical Study of the Genus *Hordeum*. Systematic and Ecogeographic Studies on Crop Genepools.

[B3] Martín A, Martínez C, Rubiales D, Ballesteros J, Guedes-Pinto H, Darvey NC (1996). *Tritordeum*: triticale's new brother cereal. Triticale: today and tomorrow.

[B4] Martín A, Martin LM, Cabrera A, Ramirez MC, Giménez MJ, Rubiales D, Hernandez P, Ballesteros J, Jaradat AA (1998). The potential of *Hordeum chilense *in breeding Triticeae species. Triticeae III.

[B5] Martín AC, Atienza SG, Ramírez MC, Barro F, Martín A (2008). Male fertility restoration of wheat in *Hordeum chilense *cytoplasm is associated with 6H^ch^S chromosome addition. Australian Journal of Agricultural Research.

[B6] Hernandez P, Dorado G, Cabrera A, Laurie DA, Snape JW, Martin A (2002). Rapid verification of wheat-*Hordeum *introgressions by direct staining of SCAR, STS, and SSR amplicons. Genome.

[B7] Hernandez P, Rubio MJ, Martin A (1996). Development of RAPD markers in tritordeum and addition lines of *Hordeum chilense *in *Triticum aestivum*. Plant Breeding.

[B8] Hernandez P, Martín A, Dorado G (1999). Development of SCARs by direct sequencing of RAPD products: a practical tool for the introgression and marker-assisted selection of wheat. Molecular Breeding.

[B9] Gale MD, Devos KM (1998). Comparative genetics in the grasses. Proceedings of the National Academy of Sciences.

[B10] Hernandez P, Hemmat M, Weeden NF, Dorado G, Martín A (1999). Development and characterization of *Hordeum chilense *chromosome-specific STS markers suitable for wheat introgression and marker-assisted selection. Theoretical and Applied Genetics.

[B11] Hernandez P, Laurie DA, Martín A, Snape JW (2002). Utility of barley and wheat simple sequence repeat (SSR) markers for genetic analysis of *Hordeum chilense *and *tritordeum*. Theoretical and Applied Genetics.

[B12] Gupta PK, Varshney RK (2000). The development and use of microsatellite markers for genetics and plant breeding with emphasis on bread wheat. Euphytica.

[B13] Varshney RK, Sigmund R, Börner A, Korzun V, Stein N, Sorrells M, Langridge P, Graner A (2005). Interspecific transferability and comparative mapping of barley EST-SSR markers in wheat, rye and rice. Plant Science.

[B14] Pinto LR, Oliveira KM, Marconi T, Garcia AAF, Ulian EC, de Souza AP (2006). Characterization of novel sugarcane expressed sequence tag microsatellites and their comparison with genomic SSRs. Plant Breeding.

[B15] Gupta PK, Rustgi S, Sharma S, Singh R, Kumar N, Balyan HS (2003). Transferable EST-SSR markers for the study of polymorphism and genetic diversity in bread wheat. Molecular Genetics and Genomics.

[B16] Decroocq V, Favé MG, Hagen L, Bordenave L, Decroocq S (2003). Development and transferability of apricot and grape EST microsatellite markers across taxa. Theoretical and Applied Genetics.

[B17] Cho YG, Ishii T, Temnykh S, Chen X, Lipovich L, McCouch SR, Park WD, Ayres N, Cartinhour S (2000). Diversity of microsatellites derived from genomic libraries and GenBank sequences in rice (*Oryza sativa *L.). Theoretical and Applied Genetics.

[B18] Boches PS, Bassil NV, Rowland LJ (2005). Microsatellite markers for *Vaccinium *from EST and genomic libraries. Molecular Ecology Notes.

[B19] Varshney RK, Graner A, Sorrells ME (2005). Genic microsatellite markers in plants: features and applications. Trends in Biotechnology.

[B20] Cordeiro GM, Casu R, McIntyre CL, Manners JM, Henry RJ (2001). Microsatellite markers from sugarcane (*Saccharum *spp.) ESTs cross transferable to erianthus and sorghum. Plant Science.

[B21] Gao L, Tang J, Li H, Jia J (2003). Analysis of microsatellites in major crops assessed by computational and experimental approaches. Molecular Breeding.

[B22] Yu JK, La Rota M, Kantety R, Sorrells M (2004). EST derived SSR markers for comparative mapping in wheat and rice. Molecular Genetics and Genomics.

[B23] Zhang LY, Ravel C, Bernard M, Balfourier F, Leroy P, Feuillet C, Sourdille P (2006). Transferable bread wheat EST-SSRs can be useful for phylogenetic studies among the Triticeae species. Theoretical and Applied Genetics.

[B24] Thiel T, Michalek W, Varshney RK, Graner A (2003). Exploiting EST databases for the development and characterization of gene-derived SSR-markers in barley (*Hordeum vulgare *L.). Theoretical and Applied Genetics.

[B25] Varshney RK, Marcel TC, Ramsay L, Russell J, Roder MS, Stein N, Waugh R, Langridge P, Niks RE, Graner A (2007). A high density barley microsatellite consensus map with 775 SSR loci. Theoretical and Applied Genetics.

[B26] Miller TE, Reader SM, Chapman V (1982). The addition of *Hordeum chilense *chromosomes to wheat. Induced variability in plant breeding. EUCARPIA Int Symp.

[B27] Kellogg EA (1998). Relationships of cereal crops and other grasses. Proceedings of the National Academy of Sciences of the United States of America.

[B28] Cabrera A, Friebe B, Jiang J, Gill BS (1995). Characterization of *Hordeum chilense *Chromosomes by C-Banding and in-Situ Hybridization Using Highly Repeated DNA Probes. Genome.

[B29] Varshney RK, Grosse I, Hahnel U, Thiel T, Rudd S, Zhang H, Prasad M, Stein N, Langridge P, Graner A (2006). Genetic mapping and physical mapping (BAC-identification) of EST-derived microsatellite markers in barley (*Hordeum vulgare *L.). Theoretical and Applied Genetics.

[B30] Murray MG, Thompson WF (1980). Rapid isolation of high molecular weight plant DNA. Nucleic Acid Research.

[B31] Hernandez P, Dorado G, Prieto P, Giménez MJ, Ramírez MC, Laurie DA, Snape JW, Martín A (2001). A core genetic map of *Hordeum chilense *and comparisons with maps of barley (*Hordeum vulgare*) and wheat (*Triticum aestivum*). Theoretical and Applied Genetics.

[B32] Jaccard P (1908). Nouvelles recherches sur la distribution florale. Bulletin de la Société Vaudoise des Sciences Naturelles.

[B33] Felsenstein J (1993). PHYLIP Phylogeny Inference Package version 3.5c. Distributed by the author.

[B34] Page RDM (1996). Tree View: An application to display phylogenetic trees on personal computers. Computer Applications in the Biosciences.

[B35] Mian RM, Saha MC, Hopkins AA, Wang Z (2005). Use of tall fescue EST-SSR markers in phylogenetic analysis of cool-season forage grasses. Genome.

[B36] Kong Q, Xiang C, Yu Z (2006). Development of EST-SSRs in *Cucumis sativus *from sequence database. Molecular Ecology Notes.

[B37] Xinquan Y, Peng L, Zongfu H, Zhongfu N, Qixin S (2005). Genetic diversity revealed by genomic-SSR and EST-SSR markers among common wheat, spelt and compactum. Progress in Natural Science.

[B38] Liu ZW, Biyashev RM, Maroof MAS (1996). Development of simple sequence repeat DNA markers and their integration into a barley linkage map. Theoretical and Applied Genetics.

[B39] Tang J, Gao L, Cao Y, Jia J (2006). Homologous analysis of SSR-ESTs and transferability of wheat SSR-EST markers across barley, rice and maize. Euphytica.

[B40] Zhang LY, Bernard M, Leroy P, Feuillet C, Sourdille P (2005). High transferability of bread wheat EST-derived SSRs to other cereals. Theoretical and Applied Genetics.

[B41] Blattner FR (2006). Multiple intercontinental dispersals shaped the distribution area of *Hordeum *(Poaceae). New Phytologist.

[B42] Komatsuda T, Tanno K, Salomon B, Bryngelsson T, von Bothmer R (1999). Phylogeny in the genus *Hordeum *based on nucleotide sequences closely linked to the vrs1 locus (row number of spikelets). Genome.

